# Enhancement of photoelectrochemical organics degradation and power generation by electrodeposited coatings of g-C_3_N_4_ and graphene on TiO_2_ nanotube arrays

**DOI:** 10.1039/c9na00437h

**Published:** 2019-09-16

**Authors:** Shuli Halevy, Eli Korin, Armand Bettelheim

**Affiliations:** Chemical Engineering Department, Ben Gurion University of the Negev Beer-Sheva 84105 Israel armandb@bgu.ac.il

## Abstract

New g-C_3_N_4_ coatings obtained *via* electropolymerization (EP) of melamine followed by a heat treatment and graphene oxide (GO) coatings based on combining GO sheets *via* EP of GO phenolic groups are used to improve the performance of photoanodes composed of TiO_2_ nanotube arrays towards the photoelectrochemical (PEC) oxidation of methanol. This process, as examined in Na_2_CO_3_ solution (pH 11.4) for the two types of coatings and serving as a model for the degradation of an organic pollutant, demonstrates enhanced PEC performance as compared to that obtained using electrochemically reduced GO coatings. PEC oxidation currents obtained with 1 M methanol reach saturation at potentials as low as ∼−0.4 V *vs.* Ag/AgCl, with the highest saturation current density of ∼2.6 mA cm^−2^ and photon-to-current efficiency of 52% as observed for the new TiO_2_NTs/g-C_3_N_4_ photoanodes. Electrochemical impedance spectroscopy measurements for these photoanodes show a charge transfer resistance one order of magnitude lower than that obtained by the other types of coatings. This indicates an enhanced charge separation ability for the photogenerated electron–hole pairs and faster interfacial charge transfer between the electron donor (methanol) and acceptor (holes). It is also demonstrated that the process of organics degradation can be achieved not only *via* an applied potential but also in a galvanic photofuelcell with methanol and oxygen serving as the fuel and oxidant, respectively. The power densities achieved with the electrochemically prepared g-C_3_N_4_ photoanodes (∼0.5 mW cm^−2^) are at least one order of magnitude higher than those reported for other TiO_2_-based systems.

## Abstract 2

Power is generated during organics photoelectrochemical degradation using electrodeposited coatings of g-C_3_N_4_ or graphene on TiO_2_ nanotube arrays.

## Graphical abstract

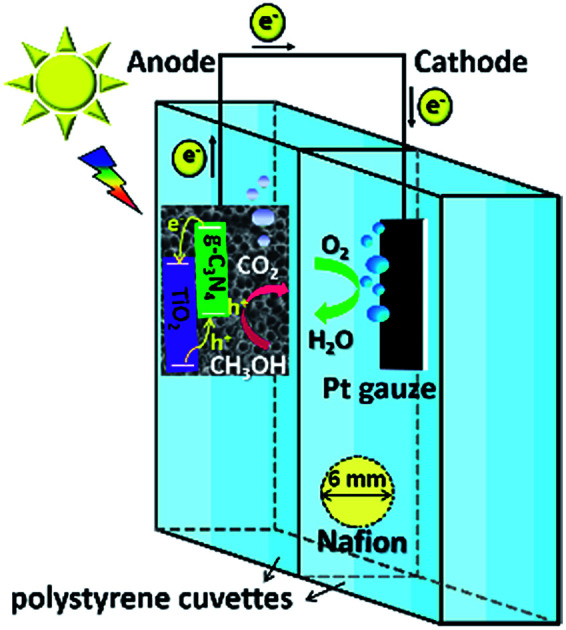

## Introduction

A serious pollution problem is caused when organic waste is discharged into our natural water sources. Therefore, it is vital to develop efficient approaches for environmental remediation. Photoelectrochemical (PEC) cells, driven by visible light, have been widely studied, since they enable organic wastewater oxidation at photoanodes with minimum energy consumption while water or protons are reduced at the cathode.^1^ Moreover, supplying oxygen (or air) to be reduced at the cathode converts the cell into a galvanic photofuelcell in which organics degradation is even accompanied by electrical power generation.^1^ To date, the low efficiency of PEC photocatalysts has limited the large scale application of such devices. One of the most widely used semiconductor photocatalysts for these applications is TiO_2_, since it has unique electronic and optical properties, high chemical stability, nontoxicity and low cost.^2^ Highly ordered TiO_2_ nanotube (NT) arrays prepared by electrochemical anodization of Ti foil were reported to show improved PEC activity compared with immobilized TiO_2_ films.^3^ Many researchers have made much effort in finding the optimal electrolyte and experimental parameters in order to efficiently achieve high quality self-organized TiO_2_NTs. Improved properties of TiO_2_NTs (optimal geometric dimensions for efficient charge transport, higher wall smoothness and tube straightness) are obtained in organic electrolytes and are responsible for improved PEC performance.^3–5^ Numerous approaches have been investigated in order to achieve a visible light response as well as suppressing electron–hole recombination, such as doping with metal ions or non-metal elements, dye sensitization, and coupling with other metal oxide semiconductors.^2^ However, recombination still limits the PEC activity of TiO_2_NTs, especially in the case of incomplete coverage as a result of decorating the surface with the above materials. Combining TiO_2_NTs with nanocarbon materials is being increasingly investigated for the enhancement of TiO_2_ PEC activity. Among the nanocarbon materials, graphene and graphitic carbon nitride (g-C_3_N_4_) are hot topics and are the most studied materials in this field nowadays.

Recently, graphitic carbon nitride (g-C_3_N_4_) as a metal-free organic semiconductor has received extensive attention, owing to its widespread potential applications in photocatalytic fields. Its attractive properties such as thermal and chemical stability, a medium (2.7 eV) band gap (BG), and a suitable band edge structure for heterojunction formation make it a perfect choice for coupling with TiO_2_.^6–8^ Graphene derivatives have also received extensive attention for coupling with TiO_2_ due to their very high electron mobility, the possibility of them acting as sensitizers by directly capturing visible light and the increased adsorption of organic compounds through π–π interactions owing to their large surface area. However, the mechanism of the PEC activity enhancement of the graphene–TiO_2_ system is still not fully understood.^9^

The preparation of g-C_3_N_4_ has been recently reviewed.^6^ In most cases g-C_3_N_4_ is synthesized by thermal condensation of nitrogen-rich precursors, such as melamine^7^ and is coupled with TiO_2_NTs *via* chemical vapor deposition (CVD)^10^ or by dip-coating of TiO_2_NTs in a g-C_3_N_4_ suspension.^11^ However, melamine exhibits a strong tendency toward sublimation during its preparation. Therefore, a considerable amount of the melamine powder is lost through the thermal process, usually conducted in a semi-closed system.^12^ Moreover, incorporating g-C_3_N_4_*via* dip-coating results in inhomogeneous surface coverage.^13^ Dip-coating from aqueous suspensions is also the main technique reported in the literature for incorporating graphene derivatives on the 3D surface of TiO_2_NTs. However, graphene sheets cover and block the top of the NTs^14^ and it is still a challenge to develop an efficient method in which homogeneous coverage and controlled coating thickness are obtained.

Electrodeposition is a good approach to immobilize coatings on the surface of 3D electrodes, owing to good stability, reproducibility, homogeneity and the possibility of controlling film thickness by adjusting the electrochemical parameters. Recently, we proposed a new and simple process consisting of melamine electropolymerization (EP) followed by a heat treatment to obtain thin, continuous and homogeneously distributed g-C_3_N_4_ films on the surface of TiO_2_NT walls.^15^ Moreover, spectroscopic characterization of these TiO_2_NTs/g-C_3_N_4_ systems indicated the possible formation of a heterojunction with a modified electronic structure.^16^

Electrodeposition of graphene has been reported to be achieved from graphene oxide (GO) suspensions at cathodic potentials, thus obtaining electro-reduced GO (erGO) films.^17,18^ Recently, we developed an alternative electrodeposition method, based upon anodic polarization of GO suspensions, which yields coatings obtained by EP of GO phenolic edge groups.^19^ The high C=C bond content is responsible for the relatively high conductivity of these epGO coatings, which is within the same order of magnitude as that of the erGO one.^20^ The EP method to obtain graphene coatings on anodized Ti seems more suitable than the one which uses cathodic polarization since the Fermi level of erGO is considered to be lower than the conduction band (CB) of TiO_2_.^21,22^ This results in a flow of electrons from the CB of TiO_2_ to that of the erGO coating in contrast to the desired opposite electron flow direction which can be expected from epGO which is characterized by a higher oxidation level.

In the present work we take advantage of the EP method for the preparation of TiO_2_NT photoanodes with nanostructured g-C_3_N_4_ as well as for GO coatings. We also demonstrate and compare the abilities of these coatings to improve the performance of TiO_2_NT photoanodes towards the PEC oxidation of methanol serving as an organic pollutant model. We show that the new electrodeposited coatings on TiO_2_NTs exhibit significantly enhanced activity towards the PEC oxidation of methanol. Higher saturation photocurrent density values, beyond 2 mA cm^−2^, are obtained compared to that of other TiO_2_/nanocarbon photoanode systems reported in the literature for methanol PEC oxidation. The improved performance of the new TiO_2_NTs/g-C_3_N_4_ photoanode is also demonstrated in a photofuelcell configuration in which methanol and oxygen serve as the fuel and oxidant, respectively.

## Experimental

TiO_2_NTs on Ti were prepared by electrochemical anodization and subsequent annealing at 500 °C for 1 hour in air.^15^ TiO_2_NTs/g-C_3_N_4_ photoanodes (geometric area: 0.9 cm^2^) were prepared *via* EP of melamine on the surface of TiO_2_NTs. The electrochemical system for EP consisted of Ti/TiO_2_NTs, Pt, and a Ag/AgCl/KCl (satd.) electrode as the working, counter and reference electrodes, respectively. After the EP process was conducted by chronoamperometry (CA) at 1.5 V, the TiO_2_NT samples were heat treated in air at 550 °C for 4 hours (heating rate: 5 °C min^−1^).^15^ TiO_2_NTs/epGO photoanodes (1 cm^2^) were fabricated *via* EP of GO on TiO_2_NTs by chronoamperometry (CA) at +1.5 V for a period of 5 min in a solution containing 0.5 mg ml^−1^ GO and 0.1 M NaHCO_3_. To obtain erGO coatings on TiO_2_NT electrodes, CA was conducted at −1.5 V for a period of 5 min in a solution containing 0.5 mg ml^−1^ GO and 0.1 M Na_2_HPO_4_.

The PEC experiments were performed with a Gamry potentiostat (series G™300) in a three-compartment glass cell using 0.1 M Na_2_CO_3_ (pH 11.4) as the electrolyte, kept at 20 °C. The photoanode faced a quartz window, through which it was illuminated (Newport Oriel Product, 200 W Hg(Xe) lamp, 100 mW cm^−2^). The counter and reference electrodes were Pt wire and a Ag/AgCl/KCl (satd.) electrode, respectively. Electrochemical impedance spectroscopy (EIS) measurements were carried out at open circuit voltage by applying a sinusoidal voltage of 10 mV and the spectra were recorded in the frequency range of 0.1 Hz to 100 kHz. Software EIS 300 (Gamry) was used for data collection and the obtained impedance plots were fitted with equivalent circuits provided by Echem Analyst (Gamry) software. The incident photon to current efficiencies (IPCEs)^23^ were measured using a 500 W Hg(Xe) arc lamp (Newport, 66142) coupled with a 1/4 m monochromator (Cornerstone 260, Newport 74125, with two gratings). Light intensity measured at each wavelength was tested using a calibrated silicon diode detector (Newport Corp. model 818-UV) to obtain the power density spectrum.

Photofuelcell experiments were conducted in a home-made cell consisting of two glued 1 cm path-length polystyrene disposable cuvettes (CVD-VIS1S, Ocean Optics), which permit the transmission of UV-visible light (*λ* > 300 nm). Holes with a diameter of 6 mm drilled on opposite walls of this compartment allowed the use of Nafion 117 (thickness ∼ 175 μm, 274674 Aldrich) as a separator between the two compartments which were filled with 0.1 M Na_2_CO_3_ solution. Measurements using this cell were performed in a two-electrode configuration. A large Pt gauze and TiO_2_NTs/g-C_3_N_4_ were used as the cathode and anode, respectively. Methanol (1 M) and oxygen served as the fuel and oxidant and were supplied to the anode and cathode, respectively.

## Results and discussion

### Characterization of the coatings on TiO_2_NTs

The microscopic and spectroscopic properties of g-C_3_N_4_ coatings obtained *via* EP on TiO_2_NT arrays have been thoroughly described in a previous report.^15^ The coatings are shown to be continuous, cover the entire TiO_2_NT inner and outer walls and are 2–3 nm thick.

**Fig. 1:**
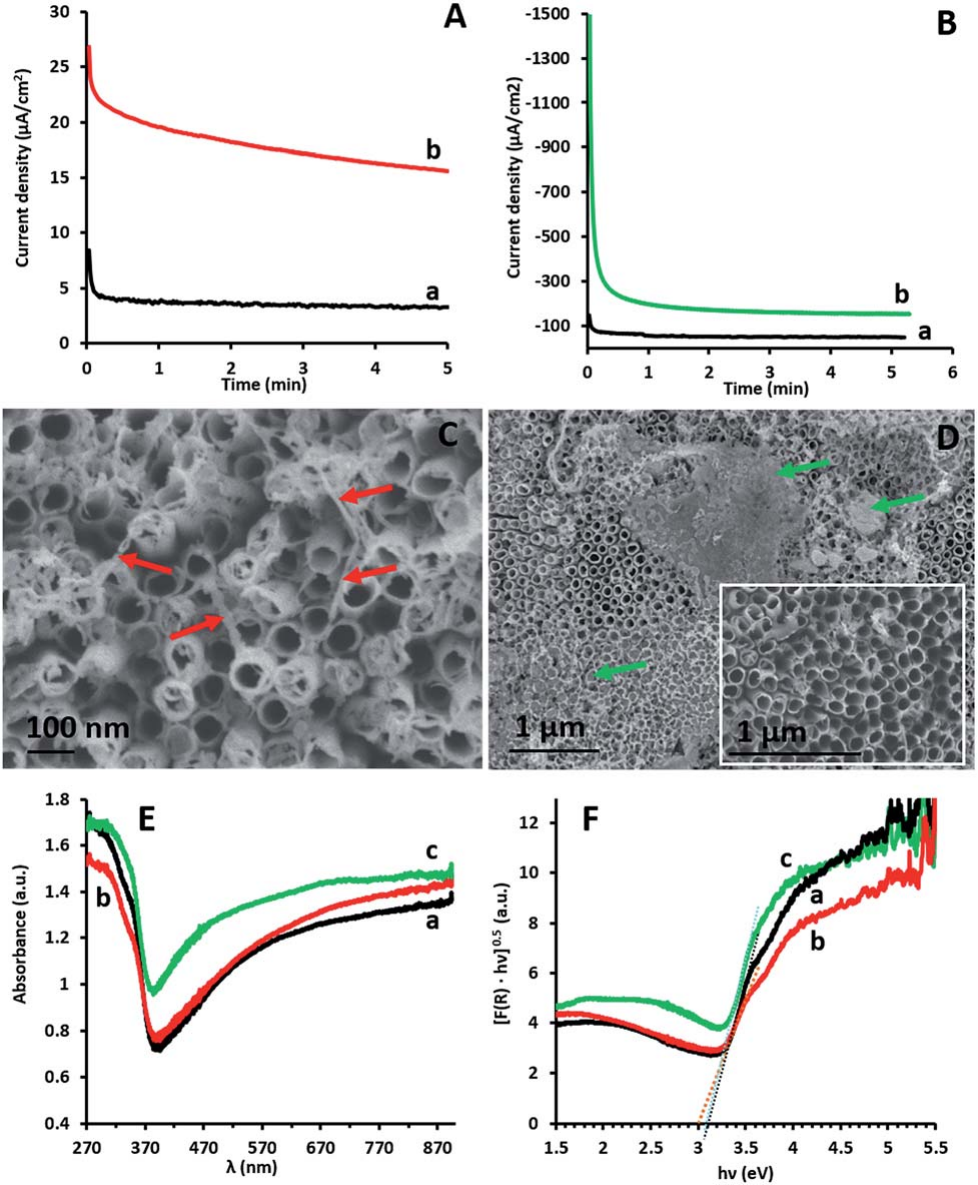
(A) CA curves obtained at +1.5 V *vs.* Ag/AgCl for the TiO_2_NT electrode in 0.1 M Na_2_CO_3_ in the absence (a) and presence (b) of 0.5 mg ml^−1^ GO. (B) CA curves obtained at −1.5 V *vs.* Ag/AgCl for the TiO_2_NT electrode in 0.1 M Na_2_HPO_4_ in the absence (a) and presence (b) of 0.5 mg ml^−1^ GO. SEM top view images for TiO_2_NTs/epGO (C) and TiO_2_NTs/erGO (D). Coating regions are marked in arrows. The inset in (D) is for bare TiO_2_NT arrays. UV-vis absorbance spectra (converted from diffuse reflectance spectra) (E) and Kubelka–Munk transformed reflectance spectra *vs.* photon energy (F) of TiO_2_NTs (a), TiO_2_NTs/epGO (b) and TiO_2_NTs/erGO (c).

Fig. 1A and B show the current density/time profiles obtained by CA during the preparation of the epGO and erGO coatings, respectively, on the TiO_2_NT electrodes. It can be seen that both for anodic and cathodic polarizations and the formation of the epGO and erGO coatings, respectively, steady state current densities are reached within the first minute after the potential has being applied. However, the steady state current density obtained for epGO coatings is one order of magnitude lower than that for the erGO ones (∼15 and 150 μA cm^−2^) respectively. This seems to indicate much thinner epGO coatings which is in accordance with our previous report (limited coating thickness of ∼30 nm on ITO electrodes) and the massive coatings with thickness of several micrometers as reported for erGO.^19^ This is also reflected in the SEM images obtained for the two types of coatings. While thin films of epGO mostly covering the outer TiO_2_ NT walls are observed for the TiO_2_NTs/epGO samples (Fig. 1C), thick coatings partially accumulated on top of the surface characterize TiO_2_NTs/erGO ones (Fig. 1D).

The optical responses of the photocatalytic films were investigated by UV-vis diffuse reflectance spectroscopy (DRS). Fig. 1E displays the absorbance spectra of uncoated TiO_2_NTs (a), TiO_2_NTs/erGO (b) and TiO_2_NTs/epGO (c). All photoanodes absorb UV light, which is characteristic of the absorption spectra of TiO_2_NTs. The broad absorption beyond 450 nm is probably caused by the trapped charge carriers in the TiO_2_NTs.^24^ The BG energies of the respective photoanodes were determined using the Kubelka–Munk transformed reflectance spectra according to the equation (*αhν*)^*n*^ = *A*(*hν* − *E*_g_), in which *α*, *h*, *ν*, and *E*_g_ are the absorption coefficient, Planck's constant, light frequency, and BG energy, respectively. The value of *n* is associated with an electronic transition,^25^ and for TiO_2_, *n* = 1/2 for the indirect transition.^16^ According to the Kubelka–Munk method the absorption coefficient is proportional to *F*(*R*) = (1 − *R*)^2^/2*R*, where *R* is the reflectance.^25^ The BG energy estimated from the intercept of the tangents to the plots of (*F*(*R*)*hν*)^1/2^*vs.* photon energy is 3.1 eV for the uncoated TiO_2_NTs and TiO_2_NTs/erGO, and 3 eV for TiO_2_NTs/epGO (Fig. 1F). Even though the BG value for the latter is just slightly lower than that of the other electrodes, the difference can stem from the ability of epGO to slightly extend the light response range of TiO_2_, which may be attributed to the formation of Ti–O–C bonds^21^ or some electronic interaction formed during the EP of GO on TiO_2_NTs. Similarly, we reported a BG reduction to a value of 2.9 eV for the TiO_2_NT photoanodes coated with g-C_3_N_4_*via* the EP process.^15^

### PEC response towards methanol oxidation of the coated TiO_2_NT photoanodes

**Fig. 2:**
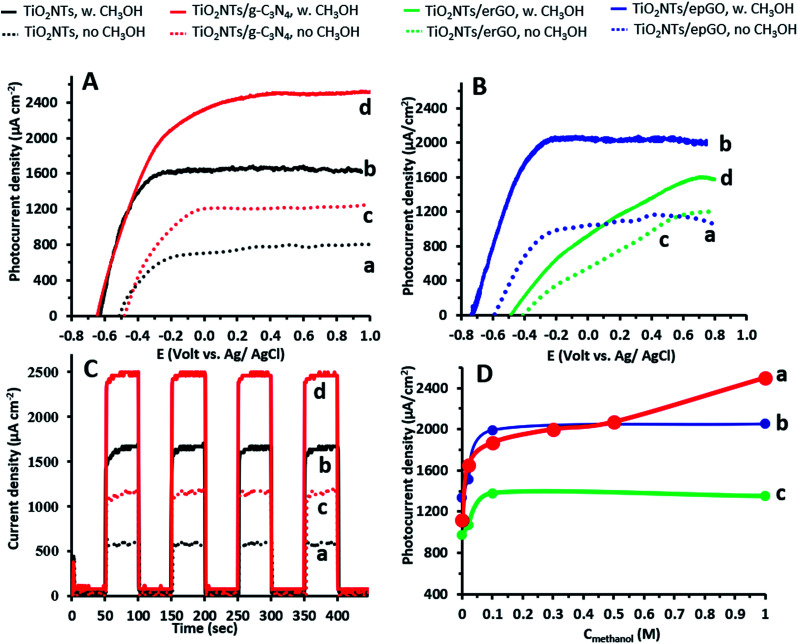
(A) Linear sweep voltammograms (5 mV s^−1^) in 0.1 M Na_2_CO_3_ for TiO_2_NT (a and b) and TiO_2_NTs/g-C_3_N_4_ (c and d) photoanodes in the absence and presence of 1 M methanol. (B) Linear sweep voltammograms (5 mV s^−1^) in 0.1 M Na_2_CO_3_ for TiO_2_NTs/epGO (a and b) and TiO_2_NTs/erGO (c and d) photoanodes in the absence and presence of 1 M methanol, respectively. (C) Current density *versus* time at a bias potential of +0.4 V *vs.* Ag/AgCl under chopped light irradiation for TiO_2_NT (a and b) and TiO_2_NTs/g-C_3_N_4_ (c and d) photoanodes in the absence and presence of 1 M methanol, respectively. (D) Photocurrent density as a function of methanol concentration at a bias of +0.4 V for TiO_2_NTs/g-C_3_N_4_ (a), TiO_2_NTs/epGO (b), and TiO_2_NTs/erGO (c) photoanodes.

Linear sweep voltammograms (LSVs) in 0.1 M Na_2_CO_3_ in the absence and presence of 1 M methanol for the TiO_2_NTs/g-C_3_N_4_ photoanode as well as for an uncoated pristine TiO_2_NT one are presented in Fig. 2A. The shape of the LSVs under illumination is typical of the n-type semiconductor behavior of the electrodes: the initial sharp increase of current reaches a nearly constant value at high potentials. The space-charge layer thickness increases with an increase in the anodic potential, until it extends through the entire wall thickness of the nanotubes. Then, further increasing the anodic potential has no effect on charge separation and a saturation photocurrent is reached.^3^ The steep rise of the photocurrent and the negative shift of the onset potential observed in the presence of methanol are typical of the PEC oxidation at various TiO_2_ photoanodes of a number of small organic molecules, including alcohols.^26–28^ The difference in potential at which saturation is reached (−0.1 and 0 V in the absence of methanol; −0.2 and +0.2 V in its presence for the uncoated and coated electrodes, respectively) can stem from the thicker walls of the TiO_2_NTs/g-C_3_N_4_ photoanode due to the presence of a continuous 2 nm g-C_3_N_4_ coating on the NTs.^15^ The saturated photocurrent density obtained for the uncoated electrode (1.7 mA cm^−2^) appears to be among the highest values reported for the PEC oxidation of methanol at TiO_2_NTs (for example: 0.7 to 1.5 mA cm^−2^).^28^ This is attributed to the difference in the anodization conditions (such as precursor concentration and applied voltage), which strongly affects TiO_2_NT properties (such as geometric dimensions and wall smoothness). These nanotube properties might be responsible for an improved e^−^–h^+^ separation and a higher diffusion length.^3–5^

LSVs for the epGO and erGO coatings obtained by similar experiments, as described above, are shown in Fig. 2B. It can be seen that both in the absence and in the presence of methanol the photocurrent densities for TiO_2_NTs/erGO are lower, and the onset potentials substantially shift anodically as compared to TiO_2_NTs/epGO. Moreover, the photocurrent density increase is sharper and saturation is reached at lower potentials for TiO_2_NTs/epGO in the presence of 1 M methanol. For example, the saturation photocurrent of TiO_2_NTs/epGO is reached at −0.25 V, which is 0.9 V more cathodic than that for TiO_2_NTs/erGO. The saturation photocurrent densities for TiO_2_NTs/g-C_3_N_4_ and TiO_2_NTs/epGO photoanodes (≈2.6 and 2.0 mA cm^−2^, respectively) are the highest among those reported for the oxidation of methanol at other TiO_2_/carbon nanostructures, such as TiO_2_ nanorods/graphene^27^ and mesoporous TiO_2_–carbon–CNT composites^29^ (0.004 and 1.0 mA cm^−2^, respectively).

CA at a potential of +0.4 V in 0.1 M Na_2_CO_3_ is demonstrated for the bare TiO_2_NT and TiO_2_NTs/g-C_3_N_4_ photoanodes in Fig. 2C. This can give valuable information regarding the presence of recombination centers and the dynamics of recombination. A steady photocurrent response is observed in the absence and presence of methanol for each switch on and off for both photoanodes. Upon starting illumination the photocurrent increases relatively slowly (5–10 s) to a constant value, since electrons further away from the back contact need some time to be collected. The absence of current transients (spikes) under illumination indicates that recombination is suppressed for both photoanodes under these conditions.^30^ Good stability of the photoanodes was deduced when testing their performance towards methanol oxidation by LSV (as in Fig. 1A) followed by CA at +0.4 V under chopped light illumination (as in Fig. 1C). Only a decay of 2.5% was observed when comparing the CA current densities under illumination before and after 5 LSV/CA cycles.

**Scheme 1:**
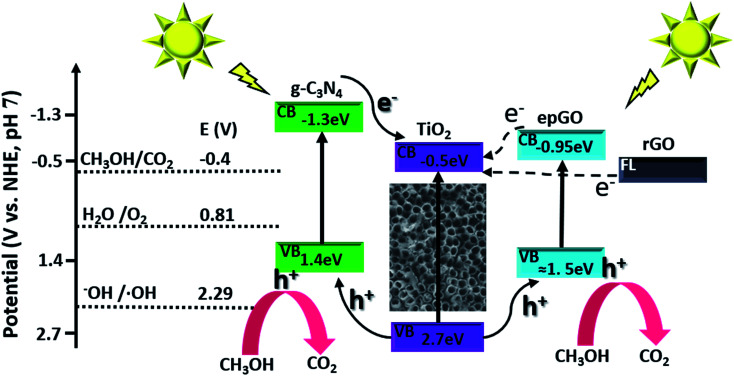
Energy diagram and schematic illustration of charge transfer in g-C_3_N_4_ and graphene coatings on TiO_2_NTs.

The dependence of steady state photocurrents on methanol concentration at a potential of +0.4 V, as obtained by CA, is depicted in Fig. 2D for bare and g-C_3_N_4_-, and erGO- and epGO-coated photoanodes (curves a, b, c, and d, respectively). The photocurrents obtained in the absence of methanol for all electrodes are due to water oxidation. The bare TiO_2_NT and g-C_3_N_4_ coated electrodes show an initial steep current increase up to a concentration of ∼0.1 M methanol and a more moderate one up to 0.3 M methanol. However, at higher concentrations, an additional increase in current is observed, which is much more significant for the TiO_2_NTs/g-C_3_N_4_ photoanode. It has been reported that for bare TiO_2_ photoanodes, the dominant oxidation mechanism at low organics concentrations is the indirect one mediated by free OH˙ radicals formed by OH^−^ oxidation.^23^ However, the direct pathway, likely to occur at higher methanol concentration, is expected to increase markedly the photocurrents due to efficient scavenging of holes in the presence of methanol and the possibility of occurrence of the current doubling effect in which two electrons are transferred to the conduction band from one photon.^1,31^ This effect is more pronounced for TiO_2_NTs/g-C_3_N_4_, since the valence band energy level of the g-C_3_N_4_ is more moderate than that of TiO_2_ (valence band energies of +1.4 and 2.7 V *vs.* NHE at pH 7, respectively^6^), thus rendering the formation of OH˙ radicals *via* oxidation of OH^−^ by holes less probable (*E*^0^ OH^−^/OH˙ = 2.29 V *vs.* NHE at pH 7 (ref. 6)). The dependence of photocurrent on methanol concentrations is similar both for erGO and epGO coatings (curves b and c): the current steeply increases up to a concentration of ∼0.1 M and reaches a constant value at higher concentrations, this value being significantly higher for the epGO than for the erGO coatings (≈2.0 and 1.4 mA cm^−2^, respectively). It also seems that the indirect rather than the direct pathway is promoted by the graphene-based coatings even at high methanol concentrations. These results can be explained by the morphology (Fig. 1) and electronic structure of the different coatings (Scheme 1). The Fermi level of rGO is lower than the CB of TiO_2_ (−0.3 and −0.5 eV, respectively),^22,32^ so electrons tend to transport from the TiO_2_ to the erGO, and therefore these electrons do not contribute to the measured photocurrent. Moreover, the rGO partial coverage diminishes the exposed active surface area of the TiO_2_NTs which is active for PEC oxidation by holes. However, the improved performances of the epGO coatings can be explained when considering the energy band levels of TiO_2_ and epGO relative to the levels of CH_3_OH and H_2_O redox potentials. It is known that GO behaves as a semiconductor and its BG is dependent on the oxygen content, with a constant CB value (≈−0.95 eV) and a VB edge, which shifts to the negative direction as the O/C ratio in GO decreases, leading to a BG range of 2.4–4.3 eV.^21^ The VB of epGO, as estimated according to that of graphite oxide^33^ with a similar oxygen content (O/C ratio of ∼0.3), is ≈1.5 eV. Although hole transfer from TiO_2_ to the epGO VB level is energetically favorable, the epGO CB level is very close to that of TiO_2_; thus electron transfer from the CB level of GO to that of TiO_2_ is less favorable than it is for the heterojunction provided by g-C_3_N_4_.^21,33^ This explains the higher methanol oxidation photocurrents measured for the g-C_3_N_4_ coatings in comparison to those for epGO ones.

**Fig. 3:**
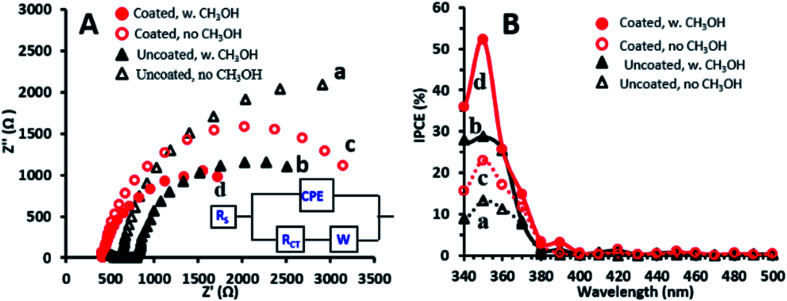
(A) EIS Nyquist plots for TiO_2_NTs (a and b) and TiO_2_NTs/g-C_3_N_4_ (c and d) in the absence and presence of 1 M methanol. Inset: equivalent circuit. (B) IPCE spectra at a bias of +0.4 V for TiO_2_NTs (a and b) and TiO_2_NTs/g-C_3_N_4_ (c and d) in the absence and presence of methanol.

Further examination was performed with the EP-obtained g-C_3_N_4_ coatings which showed the highest PEC activity towards methanol oxidation. To further understand the performance exhibited by the g-C_3_N_4_ coated photoanodes, electrochemical impedance spectroscopy (EIS) was conducted under illumination in the absence and presence of methanol for comparison of these electrodes with the uncoated ones. The EIS Nyquist plots obtained for both electrodes and solutions are characterized by semicircles (Fig. 3A), with smaller diameters reflecting lower charge transfer resistance (RCT).^34^ Both photoanodes show smaller semicircle diameters in the presence of methanol which suggests that methanol decreases RCT by improving the scavenging of holes.^31^ The Randles model, which consists of RCT and a Warburg diffusion (*W*) element in parallel with a constant phase element (CPE), fits the data obtained for the photoanodes in the presence of methanol (inset of Fig. 3A), as also reported for TiO_2_–carbon–CNT nanocomposites.^29^ The RCT values, as estimated from this model for the TiO_2_NT and TiO_2_NTs/g-C_3_N_4_ photoanodes, are 3600 and 2500 Ω cm^2^, respectively. These charge transfer resistances are one order of magnitude lower than those reported under similar conditions for TiO_2_NTs and TiO_2_NTs/g-C_3_N_4_ prepared by dip-coating of a g-C_3_N_4_ suspension.^11^ The lower charge transfer resistance of the bare TiO_2_NTs in the present research study is consistent with the PEC activity enhancement described above, and may be related to their improved properties which are very sensitive to the preparation conditions.^3–5^

The lower charge transfer resistance of the TiO_2_NTs/g-C_3_N_4_ compared to the bare TiO_2_NTs suggests that the coated photoanode exhibits enhanced charge separation ability for the photogenerated electron–hole pairs and faster interfacial charge transfer between the electron donor (methanol/water) and the electron acceptor (holes) due to the formation of a TiO_2_NTs/g-C_3_N_4_ heterojunction (Scheme 1). A good interaction between g-C_3_N_4_ and TiO_2_NTs is vital for efficient heterojunction formation,^6^ which is responsible for the improved charge transfer ability. This in turn can explain the enhanced photocurrents exhibited in the LSV and CA measurements by the g-C_3_N_4_ coated photoanode as compared to those of the uncoated ones.^34^

The incident photon to current conversion efficiency (IPCE) spectra obtained at +0.4 V (Fig. 3B) show that the IPCE values for TiO_2_NTs/g-C_3_N_4_ are higher than those for TiO_2_NTs in the absence as well as in the presence of 1 M methanol. The maximum IPCE value (52%) obtained at 350 nm in the presence of methanol is ∼1.8 times higher for TiO_2_NTs/g-C_3_N_4_ as compared to that of TiO_2_NTs (29%). This high IPCE value may imply that the coated electrode has more accessible active sites that are available for methanol oxidation. Both photoanodes show IPCE values in the presence of methanol which are twice those obtained in its absence. This indicates that the possible direct methanol oxidation by holes accompanied by the doubling effect may play an important role in the PEC process.

### Performance of the TiO_2_NTs/g-C_3_N_4_ photoanodes in a photofuelcell configuration

**Fig. 4:**
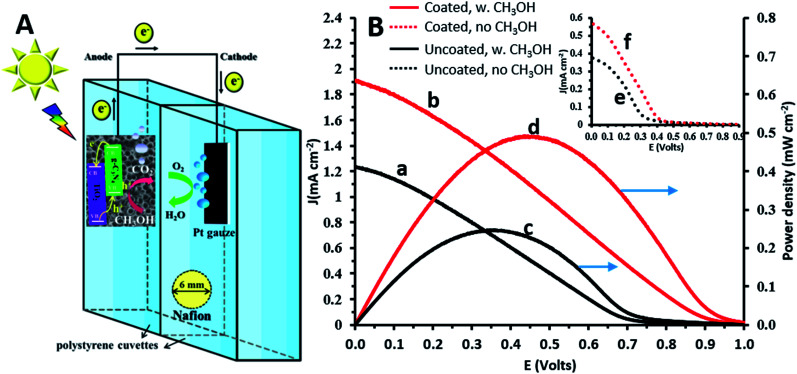
(A) Schematic illustration of a photofuelcell based on the TiO_2_NTs/g-C_3_N_4_ photoanode. (B) Current density (a and b) and power density (c and d) *vs.* voltage curves for the photofuelcell with TiO_2_NTs and TiO_2_NTs/g-C_3_N_4_ in 0.1 M Na_2_CO_3_ and 1 M methanol. Inset: current density *vs.* voltage curves under the same operating conditions without methanol for TiO_2_NTs (e) and TiO_2_NTs/g-C_3_N_4_ (f).

A photofuelcell based on a TiO_2_ photoanode with methanol and oxygen serving as the fuel and oxidant, respectively (Fig. 4A), is expected to provide a galvanic voltage of at least ∼0.8 V.^1^ This was confirmed by the measured open circuit voltages: 0.98 and 1.1 V for the illuminated TiO_2_NT and TiO_2_NTs/g-C_3_N_4_ anodes, respectively. As shown in Fig. 4B, the polarization curves obtained in the presence of methanol show appreciably higher current densities than in its absence, both for the uncoated and coated TiO_2_ photoanodes. The short circuit current densities obtained in the presence of methanol for these photoanodes, 1.2 and 1.9 mA cm^−2^ (curves a and b, respectively), are approximately threefold higher than in its absence (curves e and f, respectively). The potential of the photoanode becomes more negative in the presence of methanol, due to the more efficient hole scavenging by methanol which releases more free electrons. This raises the Fermi level, makes the semiconductor potential more electronegative,^35^ and leads to higher *E*_cell_ and current densities. The presence of the g-C_3_N_4_ coating on the TiO_2_ photoanode improves significantly the maximum power density obtained in the presence of methanol: 0.5 and 0.2 mW cm^−2^ for the coated and uncoated photoanodes (curves d and c, respectively). The power densities achieved for the electrochemically g-C_3_N_4_ coated TiO_2_ anodes are impressively higher than for similar electrodes prepared by other methods, such as TiO_2_/g-C_3_N_4_ photoanodes prepared by a sol–gel method and dip-coated on carbon fiber cloth (0.035 mW cm^−2^ with rhodamine B as the fuel^36^) and for other coatings, such as graphene/TiO_2_ nanorods prepared by a hydrothermal method (10^−3^ mWcm^−2^ with methanol as the fuel).^27^ This demonstrates the ability of the new TiO_2_/g-C_3_N_4_ systems to act not only as efficient photoanodes for the oxidation of methanol (used as a model for organic matter), but also for the generation of electricity in a photofuelcell.

## Conclusions

Incorporating carbon nanostructures on TiO_2_NT arrays *via* electrochemical methods is an efficient approach due to the possibility of obtaining homogeneous coatings with controllable thickness, which is key to an efficient PEC process. In the present work we introduce electropolymerization as a new method to electrodeposit carbon nanostructures on TiO_2_NT photoanodes. g-C_3_N_4_ and GO coatings obtained by EP show superior PEC performance towards the oxidation of methanol, serving as an organic pollutant, in comparison to that of erGO coatings obtained by the conventional cathodic electrodeposition method. The highest activity is exhibited by the TiO_2_NTs/g-C_3_N_4_ photoanodes and this is attributed to the homogeneous nature of the coatings and the formation of an efficient heterojunction which provides both enhanced charge separation ability and faster charge transfer between the electron donor (methanol) and electron acceptor (holes). These photoanodes also show a good performance in a photofuelcell configuration in which methanol and oxygen serve as the fuel and oxidant, respectively. This allows us to carry out the process not only without any required energy input but also with the generation of a relatively high power density, considering the lack of noble metals in the photocatalytic layer.^27,36,37^

## Conflicts of interest

There are no conflicts to declare.

## Supplementary Material
